# Liver Fibrosis Is Positively and Independently Associated with Leptin Circulating Levels in Individuals That Are Overweight and Obese: A FibroScan-Based Cross-Sectional Study

**DOI:** 10.3390/nu17111908

**Published:** 2025-06-01

**Authors:** Nicole Cerabino, Martina Di Chito, Davide Guido, Vincenza Di Stasi, Caterina Bonfiglio, Giuseppe Lisco, Endrit Shahini, Marianna Zappimbulso, Raffaele Cozzolongo, Valeria Tutino, Arianna Diciolla, Rosanna Mallamaci, Dolores Stabile, Anna Ancona, Sergio Coletta, Pasqua Letizia Pesole, Gianluigi Giannelli, Giovanni De Pergola

**Affiliations:** 1 Center of Nutrition for the Research and the Care of Obesity and Metabolic Diseases, National Institute of Gastroenterology “Saverio de Bellis”, IRCCS Hospital, Castellana Grotte, 70013 Bari, Italy; nicole.cerabino@irccsdebellis.it (N.C.); martina.dichito@irccsdebellis.it (M.D.C.); vincenza.distasi@irccsdebellis.it (V.D.S.); giovanni.depergola@irccsdebellis.it (G.D.P.); 2 Unit of Data Science, National Institute of Gastroenterology “Saverio de Bellis”, IRCCS Hospital, Castellana Grotte, 70013 Bari, Italy; catia.bonfiglio@irccsdebellis.it; 3 Interdisciplinary Department of Medicine, School of Medicine, University of Bari “Aldo Moro”, 70124 Bari, Italy; giuseppe.lisco@uniba.it; 4 Department of Gastroenterology, National Institute of Gastroenterology “Saverio de Bellis”, IRCCS Hospital, Castellana Grotte, 70013 Bari, Italy; endrit.shahini@irccsdebellis.it (E.S.); marianna.zappimbulso@irccsdebellis.it (M.Z.); raffaele.cozzolongo@irccsdebellis.it (R.C.); 5 Laboratory of Clinical Pathology, National Institute of Gastroenterology “Saverio de Bellis”, IRCCS Hospital, Castellana Grotte, 70013 Bari, Italy; valeria.tutino@irccsdebellis.it (V.T.); arianna.diciolla@irccsdebellis.it (A.D.); 6 Department of Biosciences, Biotechnologies and Environment, University of Bari “Aldo Moro”, Campus “E. Quagliarello”, 70126 Bari, Italy; rosanna.mallamaci@uniba.it; 7 Core Facility Biobank, National Institute of Gastroenterology “Saverio de Bellis”, IRCCS Hospital, Castellana Grotte, 70013 Bari, Italy; dolores.stabile@irccsdebellis.it (D.S.); anna.ancona@irccsdebellis.it (A.A.); sergio.coletta@irccsdebellis.it (S.C.); letizia.pesole@irccsdebellis.it (P.L.P.); 8 Scientific Direction, National Institute of Gastroenterology “Saverio de Bellis”, IRCCS Hospital, Castellana Grotte, 70013 Bari, Italy; gianluigi.giannelli@irccsdebellis.it

**Keywords:** obesity, overweight, liver fibrosis, liver steatosis, FibroScan, leptin

## Abstract

**Background:** Metabolic dysfunction-associated steatotic liver disease (MASLD) is strongly correlated with the severity of obesity, and the extent of liver fibrosis is associated with a higher risk of liver-related complications, cardiovascular events, and overall mortality. Leptin circulating levels are directly correlated with the amount of adipose tissue. **Aims:** In the present study, we investigated the association between circulating leptin levels and liver steatosis and fibrosis. **Methods:** Eighty-six patients (41.7 ± 12.6 yrs, 35 men, 41%), naïve to medications, who attended the Nutrition Center for the Research and Care of Obesity and Metabolic Diseases at the National Institute of Gastroenterology “Saverio de Bellis” for weight management, were cross-sectionally evaluated. Demographic, anthropometric, clinical, and laboratory data were collected and analyzed. All patients underwent liver ultrasonographic assessment by FibroScan to diagnose liver steatosis (controlled attenuation parameter, CAP > 275 dBm) and fibrosis (liver stiffness measurement, LSM > 8.2 kPa). **Results:** Sixty-three individuals (73.3%) had liver steatosis, and 17 (19.8%) had liver fibrosis. The mean leptin levels were 22.3 ± 14.1 ng/mL, while the BMI and waist circumference were 36.7 ± 7.2 kg/m^2^ and 114.5 ± 16.4 cm, respectively. CAP values exhibited no correlation with leptin (r = 0.09, *p* = 0.436), while a significant connection was seen between leptin and LSM (β = 0.065; *p* = 0.038). Specifically, for each unit increase in leptin, LSM values were varied by +0.065 units (*p* = 0.038). This association was independent of gender, age, insulin resistance, adiponectin, RBP4, and visfatin. This is the first study showing these results by using FibroScan assessment in patients naïve to medications. **Conclusions:** Circulating leptin concentrations are independently correlated with hepatic fibrosis in individuals with a BMI ≥ 25 kg/m^2^. These findings indicate a function for leptin in promoting liver fibrosis; however, longitudinal studies are required to elucidate the causal nature of this interaction.

## 1. Introduction

Metabolic-associated steatotic liver disease (MASLD), previously defined as non-alcoholic fatty liver disease (NAFLD) and metabolic-associated fatty liver disease (MAFLD), has emerged as the primary cause of chronic liver disease in industrialized nations, with an estimated prevalence of approximately 25% among adults [1]. MASLD represents the hepatic manifestation of metabolic syndrome and is characterized by a progressive clinical trajectory moving from simple steatosis to severe complications, including liver fibrosis, cirrhosis, and hepatocellular carcinoma [1]. Recent studies indicate that the MASLD prevalence very closely resembles that of NAFLD [2], and by 2030, it is expected that globally, 33.5% of adults may have MASLD, while systematic reviews evaluating the prevalence show that this percentage may reach 55.7% by 2040 [3].

In the context of MASLD, hepatic fibrosis serves as an important prognostic factor, regardless of the presence of steatohepatitis, and when fibrosis becomes extensive, it can lead to cirrhosis, characterized by severe liver damage [1], as several studies have established a direct relationship between the extent of fibrosis and the heightened risk of cardiovascular events and overall mortality [4,5]. As a result, the prompt and precise evaluation of fibrosis has become a vital diagnostic aim on patients with MASLD.

Among the various non-invasive techniques currently available, the transient liver elastography (such as FibroScan) provides a non-invasive estimate of liver stiffness and is extensively utilized and endorsed by international guidelines as a primary assessment tool for staging liver disease. This technique relies on the measurement of liver stiffness (LSM) and the controlled attenuation parameter (CAP) [6].

Leptin, a hormone predominantly produced by adipocytes, has multifaceted effects on regulating appetite and energy metabolism. Leptin contributes to hepatic oxidative stress and Kupffer cell activation under conditions of metabolic dysfunction, fostering inflammation and fibrosis [7]. Moreover, experimental evidence indicates that leptin can activate hepatic stellate cells to synthetize the extracellular matrix, thereby facilitating the progression of fibrosis [8]. However, clinical evidence linking circulating leptin levels to liver fibrosis in overweight or obese individuals without overt comorbidities remains scarce and often inconsistent [9].

This study aimed to assess a possible association between circulating leptin levels and ultrasound metrics of liver steatosis and fibrosis, measured with FibroScan, in a cohort of individuals that are overweight or obese and free from concomitant chronic comorbidities and pharmacological treatments.

## 2. Materials and Methods

### 2.1. Study Design and Population

This cross-sectional study was conducted at the Center for Nutrition, Research, and Care of Obesity and Metabolic Diseases, based at the National Institute of Gastroenterology “Saverio de Bellis” Research Hospital in Castellana Grotte, Bari, Italy. The study is registered on ClinicalTrials.gov with the identifier code NCT05477212. The study protocol was approved by the Local Medical Ethics Committee (Approval No. 170/CE De Bellis). All procedures involving human participants were carried out in accordance with the principles of the 1964 Declaration of Helsinki and adhered to Good Clinical Practice standards.

All participants provided written informed consent prior to enrollment in the study. Case reclamation was conducted between February 2023 and July 2024. The addition criteria comported of grown-ups progressed 18 to 65 times, a body mass index (BMI) of at least 25 kg/ m^2^, and no previous use of drugs for the condition under disquisition. Rejection criteria were established or recently diagnosed diabetes mellitus, cardiovascular diseases (CVD), respiratory insufficiency, severe gastrointestinal conditions, viral hepatitis, habitual renal failure (i.e., estimated glomerular filtration rate 20 g/ day for women), substance abuse, frailty, infectious conditions or other acute conditions affecting the situations of inflammation biomarkers, and rare metabolic diseases or mitochondrial adipose acid oxidation diseases.

Actors’ exposure to cigarette smoking and alcohol consumption was estimated. Demographic characteristics, anthropometric measures, and fasting blood samples were collected from all study participants. Additionally, all cases passed liver ultrasonographic evaluation using FibroScan within one week of registration.

### 2.2. Anthropometric Parameters

Body weight and height were assessed using a standardized measurement protocol. Participants were required to fast overnight, wear light clothing, remain barefoot, and ensure an empty bladder during the measurement. Body mass index (BMI) was calculated using the standard formula: weight in kilograms divided by height in meters squared [10]. Waist circumference (WC) was measured at the midpoint between the iliac crest and the lower edge of the ribcage and recorded in centimeters. Systolic and diastolic blood pressure values were obtained while participants were seated and at rest, using an automated device (OMRON M6). Three successive measurements were obtained, and the mean value was calculated and recorded.

### 2.3. Assessment of Liver Steatosis and Fibrosis

FibroScan has been validated as an accurate, cost-effective, and non-invasive tool for the assessment of liver steatosis and fibrosis in at-risk populations [6]. Although liver biopsy remains the gold standard for precise staging of hepatic steatosis, inflammation, and fibrosis, FibroScan provides a painless and reproducible alternative that allows for whole-liver evaluation. It is recommended by international guidelines as the first-line method for diagnosing and staging liver steatosis and fibrosis [1]. Liver steatosis was evaluated using vibration-controlled transient elastography (VCTE) with the controlled attenuation parameter (CAP) at 3.5 MHz, which estimates hepatic lipid content. Steatosis was categorized based on previously established cut-offs: ≥248 dB/m for mild (S1), ≥268 dB/m for moderate (S2), and ≥280 dB/m for severe steatosis (S3) [1]. Liver fibrosis was assessed through VCTE-derived liver stiffness measurement (VCTE-LSM). A cut-off value of ≤8 kPa was used to exclude significant fibrosis, while values ≥12 kPa were considered indicative of advanced fibrosis (grade 3) [1].

### 2.4. Laboratory Tests and Lifestyle Assessments

Blood samples were collected in the morning between 8:00 and 9:00 a.m. after an overnight fast. Serum was obtained by centrifugation and used for the measurement of fasting serum glucose (FSG), fasting insulin, triglycerides, total cholesterol, low-density lipoprotein cholesterol (LDL-C), high-density lipoprotein cholesterol (HDL-C), aspartate aminotransferase (AST), alanine aminotransferase (ALT), gamma-glutamyl transferase (γGT), uric acid, ferritin, creatinine, high-sensitivity C-reactive protein (hs-CRP), leptin, thyroid-stimulating hormone (TSH), free thyroxine (fT4), and 25-hydroxyvitamin D. All biochemical assays were performed using the COBAS 8000 modular analyzer series (Roche Diagnostics, Monza, Italy).

Leptin serum levels were determined by the ELISA assay, according to the manufacturer’s instructions (Human Leptin ELISA kit, Invitrogen, Vienna, Austria). The analytical sensitivity of the assay is <0.003 ng/mL.

Glycated hemoglobin (HbA1c) levels were estimated using the Capillarys 3 OCTA automated capillary electrophoresis system (Sebia Italia S.r.l., Bagno a Ripoli, Florence, Italy).

Insulin resistance was calculated with the homeostasis model assessment of insulin resistance (HOMA-IR) [11], a widely used index, applying the following formula: [FSG (mg/dL) × fasting serum insulin (μIU/mL)]/405.

Assessment of adiponectin was performed by using two kit: (i) MyBiosource ELISA kit (Human Adiponectin ELISA kit, San Diego, CA, USA), range: 1.563–100 ng/mL, sensitivity: 0.938 ng/mL with a sample dilution of 1:2 (42 samples); (ii) Human Adiponectin ELISA kit (Invitrogen, Vienna, Austria), with expected values in serum, range from 4 to >15 µg/mL, and a dilution of 1:2000 (44 samples). Accounting for this measurement issue, we performed a Z-score transformation, by considering the kit-related subgroups, to make comparable the values.

RBP-4 serum levels were determined by the ELISA assay, according to the manufacturer’s instructions (Human RBP-4 ELISA kit, Invitrogen, Vienna, Austria). The limit of detection of the assay is 0.034 ng/mL, and the assay range is 0.063–4 ng/mL.

Visfatin serum levels were determined by the ELISA assay, according to the manufacturer’s instructions (Human Visfatin ELISA kit, Invitrogen, Vienna, Austria). The minimum detectable dose of the assay is 1.1 ng/mL, the assay range is 1.1–300 ng/mL.

The level of physical activity was estimated using the International Physical Activity Questionnaire (IPAQ) [12].

### 2.5. Study Outcomes

The primary study outcome was to estimate the frequency of liver steatosis and fibrosis, assessed by FibroScan with a controlled attenuation parameter (CAP) and liver stiffness measurement (LSM), in our study population. The rule-in cut-off for the steatosis definition was CAP > 275 dB/m [13], and the rule-in cut-off for the fibrosis definition was LSM > 8 kPa [14]. The primary endpoint of the study was to determine the Pearson correlation coefficient (r) between controlled attenuation parameter (CAP) and leptin levels.

### 2.6. Variables of Exposure and Confounders

The exposure variable was leptin. Three potential confounding factors—gender, age, and HOMA-IR—were included in the analysis to adjust for their influence on the relationship between leptin levels and liver steatosis and fibrosis. These variables were specifically controlled for in order to refine the estimation of the association between leptin concentrations and FibroScan-derived markers, namely controlled attenuation parameter (CAP) and liver stiffness measurement (LSM).

### 2.7. Statistical Analyses

Descriptive statistics were first computed, with continuous variables summarized as the mean ± standard deviation (SD) and categorical variables presented as frequencies. Preliminary bivariate associations between FibroScan-derived markers (i.e., controlled attenuation parameter [CAP] and liver stiffness measurement [LSM]) and leptin levels, as well as other clinical parameters, were assessed using Pearson’s correlation coefficient (r).

To evaluate the independent associations between leptin and liver steatosis or fibrosis, multiple linear regression models were performed, adjusting for potential confounders, including gender, age, HOMA-IR, adiponectin, Retinol-binding protein 4 (RBP4), and visfatin. Regression coefficients (β) were used to estimate these associations. For comparative purposes, simple linear regression models were also fitted without adjustment variables to assess the change in β estimates upon removal of the covariate set. Notably, β coefficients were interpretable as expected variation of the response variable in the model, per one unit increase of leptin.

Given the relatively small sample size, Firth’s logistic regression models [15] were additionally employed to examine the relationship between leptin levels and binary outcomes of steatosis and fibrosis. These binary outcomes were defined by dichotomizing FibroScan measurements as follows: CAP > 275 dB/m (presence/absence of steatosis) and LSM > 8 kPa (presence/absence of fibrosis). Adjusted odds ratios (ORs) and 95% confidence intervals (CIs) were computed considering gender, age, HOMA-IR, adiponectin, RBP4 and visfatin as covariates.

Finally, we performed a post hoc power analysis using the Pearson’s correlation coefficients between leptin, CAP, and LSM. The two-sided Type-I-error level was set at 0.05. The post hoc power analysis is an estimate of the power of a test given the observed effect size and sample size. The underlying idea is to show that a non-significant result occurred because the power is insufficient [16]. Regarding this, to elicit all the eligible sample size values, we investigated the performance of post hoc power analysis by a simulation study, by varying the power values (x-axis) and achieving the corresponding sample sizes (y-axis), in relation to the observed effect size (Pearson’s correlation coefficients, r). However, it is worth noting that the post hoc power analysis is criticized, as has been well argued by Hoening & Heisey (2001) [17].

Of note, Pearson’s correlation coefficient (r), which was used to evaluate the raw association, is the most common effect size measure detecting the sizes of associations between two variables. r covers the whole range of relationship strengths, from no relationship whatsoever (r = 0) to a perfect linear relationship (r = 1 or r = –1) [18]. Regarding that, Cohen provided rules of thumb for interpreting these effect sizes, suggesting that an r of |0.1| represents a “small” effect size, |0.3| represents a “medium” effect size, while |0.5| represents a “large” effect size [19,20]. The post hoc power analysis is an estimate of the power of a test given the observed effect size and sample size. The underlying idea is to show that a non-significant result occurred because the power is insufficient [16].

A *p*-value of less than 0.05 was considered statistically significant, and 95% CIs were calculated. There were also reports of suggestive results (0.05 < *p* < 0.10). R software v4.3.3 [21,22], along with its packages finalfit [23], ggplot2 [24], Hmisc [25], brglm [26,27], brglm2 [27], and pwr [28], as well as StataCorp 2023 Stata Statistical Software: Release 18 (College Station, TX, USA: StataCorp LLC.), were used for all statistical analyses. Less than 5% of the data were missing, and these were imputed using the Random Forest technique via the R package missForest [29].

## 3. Results

Descriptive statistics are summarized in Table 1. Analysis was done on 86 participants (41.7 ± 12.6 years, 35 men, 41%). Of the respondents, 67 had either never smoked or had smoked in the past (78%), whereas 19 were current smokers (22%). Thirty-one men (36%) and thirty-two women (37.2%) were among the sixty-three people (73.2%) who had liver steatosis. Nine males (10.5%) and eight women (9.3%) were among the seventeen participants (19.8%) who had liver fibrosis. The average LSM and CAP values were 6.8 ± 4 kPa and 301.9 ± 64.2 dB/m, respectively. The average values of leptin were 22.3 ± 14.1 ng/mL, 22.77 ± 13.19 ng/mL in patients with steatosis and 25.47 ± 14.33 ng/mL in fibrotic ones.

Anthropometric characteristics showed that the study participants were either obese (*n* = 73, 31 males) or overweight (*n* = 13, 4 men), with mean BMI and WC of 36.7 ± 7.2 kg/m^2^ and 114.5 ± 16.4 cm, respectively. Patients with steatosis had a mean BMI and WC of 38.39 ± 6.975 kg/m^2^ and 118 ± 15.165 cm, whereas the fibrotic ones had mean values equal to 45.8 ± 8.216 kg/m^2^ and 133 ± 17.459 cm, respectively.

None had a confirmed CVD diagnosis. Other related pathological characteristics, such as a slight increase in both the diastolic and systolic arterial pressure (131.6 ± 12.5, and 82.8 ± 10.2 mmHg, respectively), were mirrored by the excess weight. Of 86 patients, 7 had incident diabetes which was not known at the enrollment time. Diabetes was diagnosed if: (i) fasting glucose levels > 125 mg/dL on two independent occasions, (ii) HbA1c >6.4% on two separate occasions, or (iii) glucose levels > 200 mg/dL following a 75 g oral glucose tolerance test (OGTT) administered over two hours. The OGTT was performed in all individuals with fasting glucose levels between 100 mg/dL and 126 mg/dL and/or HbA1c levels below 6.5%. Individuals with prediabetes were categorized within the non-diabetic category.

**Figure 1:**
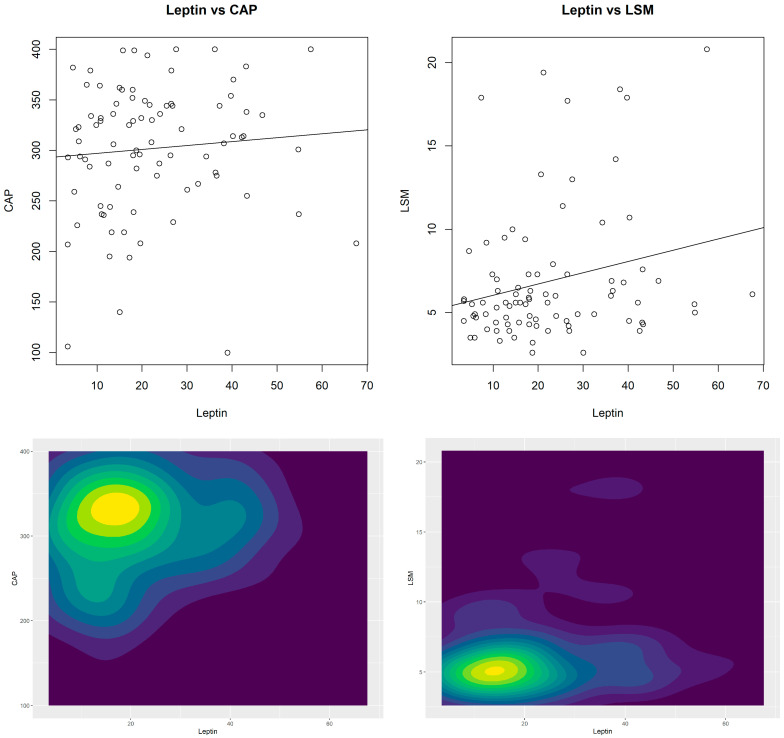
Scatter plots in the first row, contour plots in the second row: joint distributions of leptin (x-axis) and ultrasonographic features of liver steatosis (CAP) and fibrosis (LSM). In the scatter plots are also shown the regression lines.

HbA1c was 5.5 ± 0.5%, fasting serum insulin was 19.2 ± 12.3 IU/mL, and baseline FSG was 97.1 ± 12.3 mg/dL. The subjects were deemed insulin-resistant (n.v. < 2.5) if their mean HOMA-IR was 4.7 ± 3.5. All other laboratory indicators were within the normal range, with the exception of slightly lower-than-normal readings of 25-hydroxyvitamin D and a minor elevation of total and LDL-C in a group with an apparent low cardiovascular risk. Additionally, scatter plots (with a regression line) and contour plots on leptin levels and FibroScan data were derived in order to examine the joint distribution between them (Figure 1). The scatter plots report the x-y points of the patients (i.e., leptin levels-CAP or leptin levels LSM), whereas contour plots are 2D kernel density estimations of the frequency of the x–y points.

Interestingly, the distribution between leptin and CAP values is displayed on the left-side plots, while the joint distribution with LSM is displayed on the right-side plots. The slope of the regression line shows that the variables are positively associated (without taking into account the statistical inference), while the (x;y)-points in the scatter plots show the combination of values of the variables for each subject. The findings of the correlation study between circulating leptin levels and FibroScan readings are shown in Table 2. While CAP was shown to be independent (r = 0.085, *p* = 0.436; 95%CI = −0.129; 0.292), a significant direct connection was seen with LSM (r = 0.240, *p* = 0.026; 95%CI = 0.029; 0.430). The Pearson correlations with additional clinical indicators are also displayed in Table 2. However, liver steatosis (CAP; r = −0.27; *p* = 0.013) and HDL-C levels had an inverse relationship, but not fibrosis (LSM; r = −0.13; *p* = 0.229). Furthermore, we found that CAP values were strongly correlated with a number of anthropometric, clinical, and laboratory measures, including arterial pressure, insulin resistance, glucose management, liver cytotoxicity (AST and ALT), metabolic factors as RBP4 and visfatin, and weight excess (BMI and WC). For liver fibrosis, comparable associations were also discovered. Circulating leptin levels were found to be indirectly and statistically significantly correlated with both total cholesterol (r = −0.35; *p* = 0.001) and LDL-C values (r = −0.45; *p* < 0.001). Additionally, it was discovered that circulating leptin levels had a direct correlation with BMI (r = 0.49; *p* < 0.001) but a negative correlation with serum creatinine (r = −0.23; *p* = 0.034), AST (r = −0.23; *p* = 0.030), ALT (r = −0.25; *p* = 0.022), and ferritin (r = −0.22; *p* = 0.040).

Table 3 displays the findings of the regression modelling, i.e., the relationships between leptin and FibroScan data after controlling for age, gender, HOMA-IR, adiponectin, RBP4, and visfatin. For LSM, there was a significant association (β = 0.065; *p* = 0.038), but there was a trend with CAP (β = 0.817; *p* = 0.090). Interestingly, simple linear models also showed a significant association with LSM: β = 0.067; *p* = 0.026. In other terms, the expected variation (beta) of LSM values was +0.067 units (*p* = 0.026) in the simple model and +0.065 units (*p* = 0.038) in the multiple model, for a 1-unit increase in leptin. With no statistical significance, the expected variation of the CAP by the modelling was +0.817 units (*p* = 0.090) and +0.386 units (*p* = 0.436), respectively. Logistic regression models are also displayed in Table 3. In the simple models, no significant association was found (OR = 1.009, *p* = 0.620 for steatosis and OR = 1.019, *p* = 0.305 for fibrosis). However, it is interesting to note that positive relationships (OR >1) with fibrosis (ORs = 1.019, *p* = 0.451) and steatosis (ORs = 1.033, *p* = 0.212) were discovered in the multiple models, even if they were not significant. Finally, no multicollinearity issues emerged.

**Figure 2:**
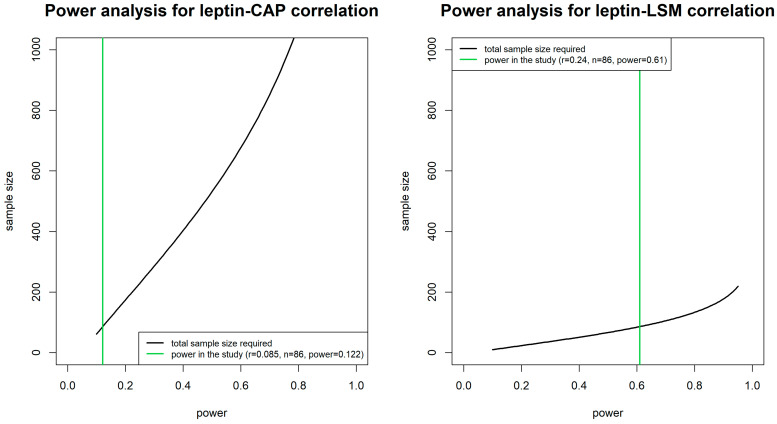
Plots of the post hoc power analysis for Pearson correlation coefficients between leptin and CAP and LSM.

Finally, post hoc analysis returned power values equal to 0.122 and 0.61 on the observed Pearson correlation coefficients of CAP (r = 0.085, *p* = 0.436) and LSM (r = 0.240, *p* = 0.026) with leptin, respectively. Of note, a simulation study was also performed to evaluate the needed sample size in relation to a power value set, to compare it with the power achieved in the study (Figure 2).

## 4. Discussion

This cross-sectional study investigated the association between circulating leptin levels and the presence of hepatic steatosis and fibrosis, as assessed by FibroScan, in a selected cohort of individuals that were overweight and obese, not in treatment with drugs, and without clinically known liver disease. Our findings demonstrate that circulating leptin levels were significantly and positively associated LSM (β = 0.070; *p* = 0.024), even after adjustment for age, gender, and insulin resistance (HOMA-IR), whereas the association with steatosis (as measured by CAP) was only marginally significant (β = 0.901; *p* = 0.061).

These data suggest that leptin may play an active role in hepatic fibrogenesis, in addition to its involvement in intrahepatic lipid metabolism, supporting the hypothesis that this adipokine acts as a mediator within the metabolic–inflammatory–fibrotic continuum of MASLD [1,30]. The independent association between leptin and LSM was also confirmed in univariate linear regression analyses (β = 0.067; *p* = 0.026), highlighting a potential direct effect of the adipokine on hepatic remodeling. Indeed, leptin is known to activate the JAK2/STAT3 and MAPK signaling pathways in hepatocytes and hepatic stellate cells, promoting TGF-β1 production and type I and III collagen deposition [31,32,33]. Preclinical studies further support the role of leptin as a driver of the epithelial-mesenchymal transition (EMT), a key step in fibrotic progression [34].

Conversely, no significant correlation was observed between leptin and CAP, a marker of hepatic fat content (r = 0.09; *p* = 0.436), in either univariate or multivariate regression models. This finding is in contrast with earlier studies identifying leptin as a biomarker of hepatic steatosis in individuals with obesity and the metabolic syndrome [35], but in alignment with more recent evidence suggesting that intrahepatic lipid accumulation is more strongly influenced by insulin resistance, dyslipidemia, and dietary factors than by leptin per se [36].

Furthermore, leptin was positively associated with the BMI (r = 0.49; *p* < 0.001), consistently with the fact that leptin synthesis is proportional to the total adipose mass [37].

All these findings suggest that leptin is a potential biomarker of hepatic fibrosis in patients with obesity. Unlike conventional biochemical markers (ALT, AST, γGT), which often remain within normal ranges during the early stages of MASLD, leptin may be an earlier risk factor that could be integrated into multifactorial predictive algorithms.

### Strengths and Limitations

This study has a number of advantages that improve its clinical relevance and methodological soundness.

Firstly, the inclusion of a well-characterized population composed of overweight and obese treatment-naïve individuals with no diagnosed or known chronic liver diseases and evaluated in a real-world clinical setting for cardiovascular prevention allows the investigation of the association between leptin and liver injury in a subclinical stage—a phase that has been relatively underexplored. This approach enables a more accurate isolation of leptin’s effects from potential confounding factors related to pharmacological treatments, comorbidities, or established metabolic disorders [1,30].

Another key strength is the FibroScan use, a validated and non-invasive diagnostic tool, which enables accurate and reproducible quantification of hepatic steatosis and fibrosis.

The combined use of correlation analyses, multiple regression models, and penalized logistic models (Firth regression) represents an additional methodological strength. In particular, the latter approach allows for more stable and less biased estimates in settings characterized by small sample sizes or imbalanced outcome distributions [27,38]. Importantly, the study was adequately controlled for major known metabolic confounders—age, gender, and insulin resistance—which improves the internal validity of the findings and supports the hypothesis of an independent association between leptin and hepatic fibrosis [27].

However, some limitations should also be acknowledged. Firstly, the cross-sectional observational design prevents establishing causal relationships between leptin and liver fibrosis, and does not allow causal inferences to be drawn; it remains possible that hyperleptinemia is a consequence rather than a determinant of liver fibrosis. Longitudinal or prospective studies will therefore be necessary to clarify the directionality and temporality of the association [39].

Secondly, the relatively small sample size (*n* = 86), along with the low prevalence of significant hepatic fibrosis (19.8%), may have limited the statistical power of the analyses, particularly regarding the secondary outcomes. This is supported by the results of the post hoc analysis, which revealed limited power in detecting the correlation between leptin and CAP [17]. Hence, increasing the sample size in future studies, especially for the fibrosis group, could ensure more reliable detection of associations.

In addition, a control group of healthy patients was missing. Concerning that, it is worth it to point out that given the cross-sectional observational (i.e., not interventional) nature of the study, it was carried out in a specific group of individuals that were overweight and obese, not in treatment with drugs, and without clinically known liver disease.

Moreover, since the study is observational, not interventional, we prefer not to provide suggestions on the effects of the diet on both leptin levels and liver health, nor even on validated dietary questionnaires or biomarkers of dietary intake. We just shed a light in an explorative way on the association between levels and fibrosis and steatosis, by adjusting for gender, age, HOMA, adiponectin, RBP4, and visfatin.

Finally, it is worth it to point out that the assessment of adiponectin has been performed by using two kits. However, since different types of kits (and reference values) are used in the literature, to our knowledge, no technical concerns (i.e., measurement bias) emerged, although it would probably be necessary to make an agreement analysis between commercially available kits to compare the data.

In summary, despite some inherent limitations related to the study design and sample size, our data provide original and clinically relevant insights into the relationship between leptin and liver injury, laying the groundwork for prospective and interventional studies aimed at validating leptin as a potential early, non-invasive biomarker of fibrotic progression.

## 5. Conclusions

In an apparently healthy overweight or obese population, our study offers early evidence of a correlation between circulating leptin levels and hepatic fibrosis, independent of age, gender, and insulin resistance. To our knowledge, it specifically addresses a notable gap in the literature regarding the role of leptin in early stage liver fibrosis among overweight and obese individuals. In contrast, no robust association was observed with hepatic steatosis, suggesting a potentially more specific role for leptin in hepatic fibrogenesis rather than intrahepatic lipid accumulation.

These findings support the hypothesis that leptin may serve as an early and potentially predictive marker of liver fibrosis in patients with MASLD, even in individuals without overt liver disease. The identification of such an association through a non-invasive tool (FibroScan) lays the foundation for future strategies aimed at early risk stratification and intervention and hence has implications on both cardiovascular and liver disease prevention.

Large-scale longitudinal studies and functional assessments of leptin will be required to gain a better definition of the precise pathogenic and prognostic role of this hormone in the progression of metabolic liver disease.

## Figures and Tables

**Table 1:** Background characteristics of the study population.

	Overall (*n* = 86)
Ultrasonographic measures of liver steatosis and fibrosis *	
FibroScan CAP (dB/m)	301.9 ± 64.2
FibroScan LSM (kPa)	6.87 ± 4.0
Steatosis * (yes/no; *n* and %)	63/23 (73.3%/26.7%)
Fibrosis * (yes/no; *n* and %)	17/69 (19.8%/80.2%)
Variable of exposure	
Leptin (ng/mL)	22.32 ± 14.12
Hallmarks of glucose control and insulin resistance	
FSG (mg/dL)	97.1 ± 12.3
HbA1c (%)	5.5 ± 0.5
Fasting serum insulin (U/mL)	19.2 ± 12.5
HOMA-IR (score)	4.7 ± 3.5
Diabetes (yes/no)	7/79 (8.1%/91.9%)
Demographic and lifestyle characteristics	
Age (yrs)	41.7 ± 12.6
Gender (male/female; *n* and %)	35/51 (41%/59%)
Current smokers (yes/no; *n* and %)	19/67 (22%/78%)
IPAQ (score)	1809.3 ± 1566.9
Anthropometric and clinical parameters	
BMI (kg/m^2^)	36.7 ± 7.2
Waist circumference (cm)	114.5 ± 16.4
Systolic blood pressure (mmHg)	131.6± 12.52
Diastolic blood pressure (mmHg)	82.80± 10.24
Laboratory tests	
Triglycerides (mg/dL)	120.57± 71.67
HDL cholesterol (mg/dL)	51.53 ± 14.70
LDL cholesterol (mg/dL)	137.6 ± 33.01
Total cholesterol (mg/dL)	203.7 ± 41.13
TSH (µmU/mL)	1.96 ± 1.31
FT3 (pg/mL)	3.36 ± 0.40
FT4 (ng/dL)	10.92 ± 1.96
25-hydroxyvitamin D (ng/mL)	19.95 ± 6.73
Uric acid (mg/dL)	5.35 ± 1.23
Creatinine (mg/dL)	0.80 ± 0.14
AST (U/L)	23.85 ± 13.14
ALT (U/L)	35.21 ± 28.76
γGT (U/L)	28.44 ± 19.34
High-sensitive C-reactive protein (mg/dL)	1.14 ± 5.75
Ferritin (ng/mL)	149.2 ± 163.84
Adiponectin (u)	0.00 ± 1
RBP4 (μg/mL)	29.71 ± 29.84
Visfatin (ng/mL)	2.15 ± 1.63

Abbreviations: BMI: Body Mass Index; CAP, Controlled Attenuation Parameter; LSM, Liver Stiffness Measurement; FSG, Fasting Serum Glucose; HbA1c, Glycated hemoglobin; HOMA-IR, Homeostasis Model Assessment–Insulin Resistance; IPAQ, International Physical Activity Questionnaire; HDL, High-Density Lipoprotein; LDL, Low-Density Lipoprotein; TSH, Thyroid-Stimulating Hormone; FT4, Free Tetraiodothyronine; FT3, Free Triiodothyronine; AST, Aspartate amino transferase; ALT, Alanine amino transferase; γGT, gamma Glutamyl-transferase; u, Standard Unit; RBP4, Retinol-binding protein 4. * Steatosis and fibrosis were diagnosed in the presence of CAP >275 dB/m and LSM >8 kPa, respectively.

**Table 2:** Pearson correlations of CAP, LSM, and leptin with anthropometrical, clinical, and laboratory parameters.

	CAP r (*p*-Value)	LSM r (*p*-Value)	Leptin r (*p*-Value)
LSM	**0.36 (0.001)**	-	-
Leptin	0.09 (0.436)	**0.24 (0.026)**	-
FSG	**0.23 (0.033)**	**0.24 (0.026)**	−0.07 (0.526)
HbA1c	**0.34 (0.002)**	**0.36 (<0.001)**	0.09 (0.389)
Insulin	**0.50 (<0.001)**	**0.47 (<0.001)**	0.18 (0.104)
HOMA-IR	**0.49 (<0.001)**	**0.50 (<0.001)**	0.14 (0.187)
IPAQ	−0.15 (0.154)	−0.03 (0.762)	−0.15 (0.154)
BMI	**0.48 (<0.001)**	**0.62 (<0.001)**	**0.49 (<0.001)**
WC	**0.59 (<0.001)**	**0.48 (<0.001)**	*0.20 (0.066)*
Systolic blood pressure	**0.21 (0.047)**	0.17 (0.110)	0.05 (0.653)
Diastolic blood pressure	**0.35 (0.001)**	0.15 (0.162)	0.08 (0.472)
Triglycerides	**0.33 (0.002)**	0.12 (0.272)	−0.17 (0.120)
HDL cholesterol	**−0.27 (0.013)**	−0.13 (0.229)	*0.18 (0.094)*
LDL cholesterol	−0.05 (0.630)	*−0.21 (0.055)*	**−0.45 (<0.001)**
Total cholesterol	−0.14 (0.208)	−0.17 (0.108)	**−0.35 (0.001)**
TSH	**0.22 (0.042)**	0.00 (0.991)	0.10 (0.359)
FT3	0.08 (0.479)	−0.03 (0.807)	0.03 (0.811)
FT4	**0.23 (0.030)**	0.08 (0.446)	−0.07 (0.526)
25-hydroxyvitamin D	−0.06 (0.560)	0.00 (0.969)	−0.11 (0.322)
Uric acid	**0.42 (<0.001)**	*0.19 (0.083)*	*−0.20 (0.064)*
Creatinine	*0.21 (0.054)*	0.04 (0.723)	**−0.23 (0.034)**
AST	**0.32 (0.002)**	**0.38 (<0.001)**	**−0.23 (0.030)**
ALT	**0.39 (<0.001)**	**0.32 (0.003)**	**−0.25 (0.022)**
γGT	**0.35 (0.001)**	**0.29 (0.007)**	*−0.21 (0.054)*
Ferritin	**0.39 (<0.001)**	*0.20 (0.063)*	**−0.22 (0.040)**
Adiponectin	−0.04 (0.744)	−0.07 (0.494)	0.04 (0.681)
RBP4	**0.22 (0.046)**	0.03 (0.788)	−0.07 (0.494)
Visfatin	**−0.34 (0.001)**	**−0.23 (0.031)**	−0.02 (0.835)

Abbreviations: CAP, Controlled attenuation parameter; LSM, Liver stiffness measurement; FSG, Fasting serum glucose; HbA1c, Glycated hemoglobin; HOMA-IR, Homeostasis model assessment–insulin resistance; IPAQ, International Physical Activity Questionnaire; BMI, Body mass index; WC, Waist circumference; HDL, High-density lipoprotein; LDL, Low-density lipoprotein; TSH, Thyroid-stimulating hormone; FT4, Free tetraiodothyronine; FT3, Free triiodothyronine; AST, Aspartate amino transferase; ALT, Alanine amino transferase; γGT, gamma Glutamyl-transferase; RBP4, Retinol-binding protein 4. r: Pearson correlation coefficient. In **bold**, significant results (*p* < 0.05), in *italics* (0.05 < *p* < 0.10) trends.

**Table 3:** Results of the regression analysis of the associations between FibroScan measurements and leptin levels.

	β of Leptin on FibroScan CAP	β of Leptin on FibroScan LSM	OR of Leptin on Steatosis	OR of Leptin on Fibrosis
	Linear Model	Linear Model	Logistic Models	Firth’s Logistic Models
**Ordinary model**	β = 0.386 *p* = 0.436 95%CI = −0.596; 1.369	**β= 0.067** ***p* = 0.026** **95%CI = 0.008; 0.127**	OR = 1.009 *p* = 0.620 95%CI = 0.974; 1.045	OR = 1.019 *p* = 0.305 95%CI = 0.982; 1.056
Multiple model *	*β = 0.817* *p = 0.090* *95%CI = −0.127; 1.761*	**β = 0.065** ***p* = 0.038** **95%CI = 0.004; 0.127**	OR = 1.025 *p* = 0.345 95%CI = 0.974; 1.078	OR = 1.015 *p* = 0.533 95%CI = 0.967; 1.066

Abbreviations: CAP, Controlled attenuation parameter; LSM, Liver stiffness measurement; β, Linear regression coefficient, i.e., expected variation of the response variable in the model, per one unit increase of leptin. OR, Odds ratio. *p*, *p*-value. In **bold,** significant results (*p* < 0.05), in *italics* (0.05 < *p* < 0.10) trends; 95%CI: 95% confidence intervals. * Adjusted for gender, age, HOMA, adiponectin, RBP4, and visfatin. Results of the regression analysis on the associations between FibroScan measurements (outcomes) with leptin levels: the columns present the results returned by the models in terms of association measures (i.e., β or OR), the rows present the modelling nature.

## Data Availability

The original contributions presented in this study are included in the article. Further inquiries can be directed to the corresponding author. The data presented in this study aren’t publicly available due to sensitive data concerns. However, data sharing will be evaluated upon request to the corresponding author.

## Abbreviations

The following abbreviations are used in this manuscript:

MASLD	Metabolic Dysfunction-Associated Steatotic Liver Disease
CVD	Cardiovascular Diseases
BMI	Body Mass Index
WC	Waist Circumference
CAP	Controlled Attenuation Parameter
LSM	Liver Stiffness Measurement
FSG	Fasting Serum Glucose
LDL-C	Low-Density Lipoprotein Cholesterol
HDL-C	High-Density Lipoprotein Cholesterol
AST	Aspartate-Amino Transferase
ALT	Alanine-Amino Transferase
γGT	Gamma Glutaminyl-Transferase
HbA1c	Glycated Hemoglobin
HOMA-IR	Homeostasis Model Assessment of Insulin Resistance
IPAQ	International Physical Activity Questionnaire

## References

[1] European Association for the Study of the Liver (EASL), European Association for the Study of Diabetes (EASD), European Association for the Study of Obesity (EASO) (2024). EASL–EASD–EASO Clinical Practice Guidelines on the management of MASLD. J. Hepatol..

[2] Ciardullo S., Carbone M., Invernizzi P., Perseghin G. (2023). Exploring the Landscape of Steatotic Liver Disease in the General US Population. Liver Int..

[3] Kalligeros M., Vassilopoulos A., Vassilopoulos S., Victor D.W., Mylonakis E., Noureddin M. (2023). Prevalence of Steatotic Liver Disease (MASLD, MetALD, and ALD) in the United States: NHANES 2017–2020. Clin. Gastroenterol. Hepatol..

[4] Targher G., Byrne C.D. (2021). MASLD and increased risk of cardiovascular events. Nat. Rev. Cardiol..

[5] Dulai P.S., Singh S., Patel J., Soni M., Prokop L.J., Younossi Z., Sebastiani G., Ekstedt M., Hagstrom H., Nasr P. (2017). Increased risk of mortality by fibrosis stage in NAFLD: Systematic review and meta-analysis. Hepatology.

[6] Eddowes P.J., Sasso M., Allison M., Tsochatzis E., Anstee Q.M., Sheridan D., Guha I.N., Cobbold J.F., Deeks J.J., Paradis V. (2019). Accuracy of FibroScan Controlled Attenuation Parameter and Liver Stiffness Measurement in Assessing Steatosis and Fibrosis in Patients With Nonalcoholic Fatty Liver Disease. Gastroenterology.

[7] Chatterjee S., Ganini D., Tokar E.J., Kumar A., Das S., Corbett J., Kadiiska M.B., Waalkes M.P., Diehl A.M., Mason R.P. (2013). Leptin is key to peroxynitrite-mediated oxidative stress and Kupffer cell activation in experimental non-alcoholic steatohepatitis. J. Hepatol..

[8] Wang J., Leclercq I., Brymora J.M., Xu N., Ramezani-Moghadam M., London R.M., Brigstock D., George J. (2009). Kupffer cells mediate leptin-induced liver fibrosis. Gastroenterology.

[9] Polyzos S.A., Kountouras J. (2016). Leptin in NAFLD: A friend or a foe?. Hepatol. Res..

[10] Nuttall F.Q. (2015). Body Mass Index: Obesity, BMI, and Health: A Critical Review. Nutr. Today.

[11] Matthews D.R., Hosker J.P., Rudenski A.S., Naylor B.A., Treacher D.F., Turner R.C. (1985). Homeostasis model assessment: Insulin resistance and beta-cell function from fasting plasma glucose and insulin concentrations in man. Diabetologia.

[12] Craig C.L., Marshall A.L., Sjöström M., Bauman A.E., Booth M.L., Ainsworth B.E., Pratt M., Ekelund U., Yngve A., Sallis J.F. (2003). International physical activity questionnaire: 12-country reliability and validity. Med. Sci. Sports Exerc..

[13] Berzigotti A., Tsochatzis E., Boursier J., Castera L., Cazzagon N., Friedrich-Rust M., Petta S., Thiele M., European Association for the Study of the Liver (2021). EASL Clinical Practice Guidelines on non-invasive tests for evaluation of liver disease severity and prognosis—2021 update. J. Hepatol..

[14] Expert Panel on Liver Stiffness Measurement (2014). Clinical Application of Transient Elastography in the Diagnosis of Liver Fibrosis: An Expert Panel Review and Opinion. J. Clin. Transl. Hepatol..

[15] Wang X. (2014). Firth logistic regression for rare variant association tests. Front. Genet..

[16] Crespi C.M. (2020). Power and Sample Size in R.

[17] Hoening J.M., Heisey D.M. (2001). The Abuse of Power: The Pervasive Fallacy of Power Calculations for Data Analysis. Am. Stat..

[18] Shoukri M.M. (2018). Analysis of Correlated Data with SAS and R.

[19] Cohen J. (1988). Statistical Power Analysis for the Behavioral Sciences.

[20] Cohen J. (1990). Things I have learned (so far). Am. Psychol..

[21] Stekhoven D.J. MissForest: Nonparametric Missing Value Imputation Using Random Forest, R Package Version 1.5. https://CRAN.R-project.org/package=missForest.

[22] Stekhoven D.J., Buehlmann P. (2012). MissForest—Non-parametric missing value imputation for mixed-type data. Bioinformatics.

[23] Harrison E., Drake T., Pius R. (2024). Finalfit: Quickly Create Elegant Regression Results Tables and Plots when Modelling. R Package Version 1.0.8. https://CRAN.R-project.org/package=finalfit.

[24] Wickham H. (2016). ggplot2: Elegant Graphics for Data Analysis.

[25] Harrell J.R., Hmisc: Harrell Miscellaneous (2024). R Package Version 5.2-0. https://CRAN.R-project.org/package=Hmisc.

[26] Kosmidis I., Brglm: Bias Reduction in Binary-Response Generalized Linear Models (2021). R Package Version 0.7.2. https://cran.r-project.org/package=brglm.

[27] Kosmidis I., Firth D. (2021). Jeffreys-prior penalty, finiteness and shrinkage in binomial-response generalized linear models. Biometirka.

[28] Champely S., Pwr: Basic Functions for Power Analysis (2020). R Package Version 1.3-0. https://CRAN.R-project.org/package=pwr.

[29] R Core Team (2024). A Language and Environment for Statistical Computing. R Foundation for Statistical Computing.

[30] Tilg H., Moschen A.R. (2010). Evolution of inflammation in nonalcoholic fatty liver disease: The multiple parallel hits hypothesis. Hepatology.

[31] Marra F. (2002). Leptin and liver fibrosis: A matter of fat. Gastroenterology.

[32] Saxena N.K., Yang Y., Floyd J., Anania F.A. (2004). Leptin promotes the development of hepatic fibrosis in a rodent model of nonalcoholic steatohepatitis. Hepatology.

[33] Saxena N.K., Ikeda K., Rockey D.C., Friedman S.L., Anania F.A. (2002). Leptin in hepatic fibrosis: Evidence for increased collagen production in stellate cells and lean littermates of ob/ob mice. Hepatology.

[34] Stefanou N., Papanikolaou V., Furukawa Y., Nakamura Y., Tsezou A. (2010). Leptin as a critical regulator of hepatocellular carcinoma development through modulation of human telomerase reverse transcriptase. BMC Cancer.

[35] Polyzos S.A., Aronis K.N., Kountouras J., Raptis D.D., Vasiloglou M.F., Mantzoros C.S. (2016). Serum leptin in nonalcoholic fatty liver disease: A systematic review and meta-analysis. Diabetologia.

[36] Machado M.V., Cortez-Pinto H. (2009). Insulin resistance and steatosis in chronic hepatitis C. Ann. Hepatol..

[37] Considine R.V., Sinha M.K., Heiman M.L., Kriauciunas A., Stephens T.W., Nyce M.R., Ohannesian J.P., Marco C.C., McKee L.J., Bauer T.L. (1996). Serum immunoreactive-leptin concentrations in normal-weight and obese humans. N. Engl. J. Med..

[38] Lisco G., Guido D., Cerabino N., Di Chito M., Donvito R., Bonfiglio C., Shahini E., Zappimbulso M., Randazzo C., Barletta D. (2025). Liver steatosis is positively associated with plasminogen activator inhibitor-1 in apparently healthy individuals with overweight and obesity: A FibroScan-Based Cross-Sectional study. J. Transl. Med..

[39] Friedman S.L. (2003). Liver fibrosis—From bench to bedside. J. Hepatol..

